# Management of Coronary Vulnerable Plaques: A Focus on Preventive Percutaneous Coronary Intervention

**DOI:** 10.31083/RCM26712

**Published:** 2025-04-17

**Authors:** Stefano Rigattieri, Marco Redivo, Matteo Casenghi, Marta Belmonte, Francesca Giovannelli, Antonella Tommasino, Emanuele Barbato, Andrea Berni

**Affiliations:** ^1^Cardiology Division, Sant’Andrea University Hospital, 00189 Rome, Italy; ^2^Department of Advanced Biomedical Sciences, University Federico II, 80138 Naples, Italy; ^3^Department of Clinical and Molecular Medicine, Sapienza University of Rome, 00185 Rome, Italy

**Keywords:** vulnerable plaque, percutaneous coronary intervention, coronary thrombosis

## Abstract

Vulnerable or high-risk coronary plaques are usually referred to as angiographically mild to moderate lesions characterized by a large plaque burden, positive vessel remodeling, thin fibrous cap, and large necrotic/lipid core. According to several pathology studies, these plaques represent the substrate of coronary thrombosis in about two-thirds of cases; therefore, there has been increasing interest in detecting and treating vulnerable plaques (VPs). Nowadays, VP detection is possible through noninvasive and invasive imaging techniques, such as coronary computed tomography, magnetic resonance imaging, intravascular ultrasound, optical coherence tomography, and near-infrared spectroscopy. Since VPs were shown to be associated with cardiovascular events in observational studies, pharmacological and non-pharmacological strategies have been investigated to achieve a regression and/or a passivation of these plaques. In addition to pharmacological therapies, mainly focused on lipid-lowering agents, there has been a recent growing interest in interventional therapies, including coronary scaffolds, stents, and drug-coated balloons. This led to the concept of preventive percutaneous coronary intervention, which, unlike the treatment of culprit lesions in acute coronary syndromes or of ischemia-inducing stenoses, as recommended by guidelines, implies the treatment of angiographically and functionally non-significant lesions based on one or more high-risk plaque characteristics as identified by noninvasive or intracoronary imaging. This article provides an updated review of key concepts in defining and detecting VPs; their prognostic value and available pharmacological and interventional management evidence will also be discussed.

## 1. Introduction

Cardiovascular diseases (CVDs) are the leading cause of death worldwide, with almost 18 million people dying from CVDs in 2019, one-third of all global deaths. Of these, 85% were attributable to heart attack and stroke. Furthermore, 38% of all premature deaths in people younger than 70 years in 2019 were caused by CVDs [1].

**Fig. 1.**
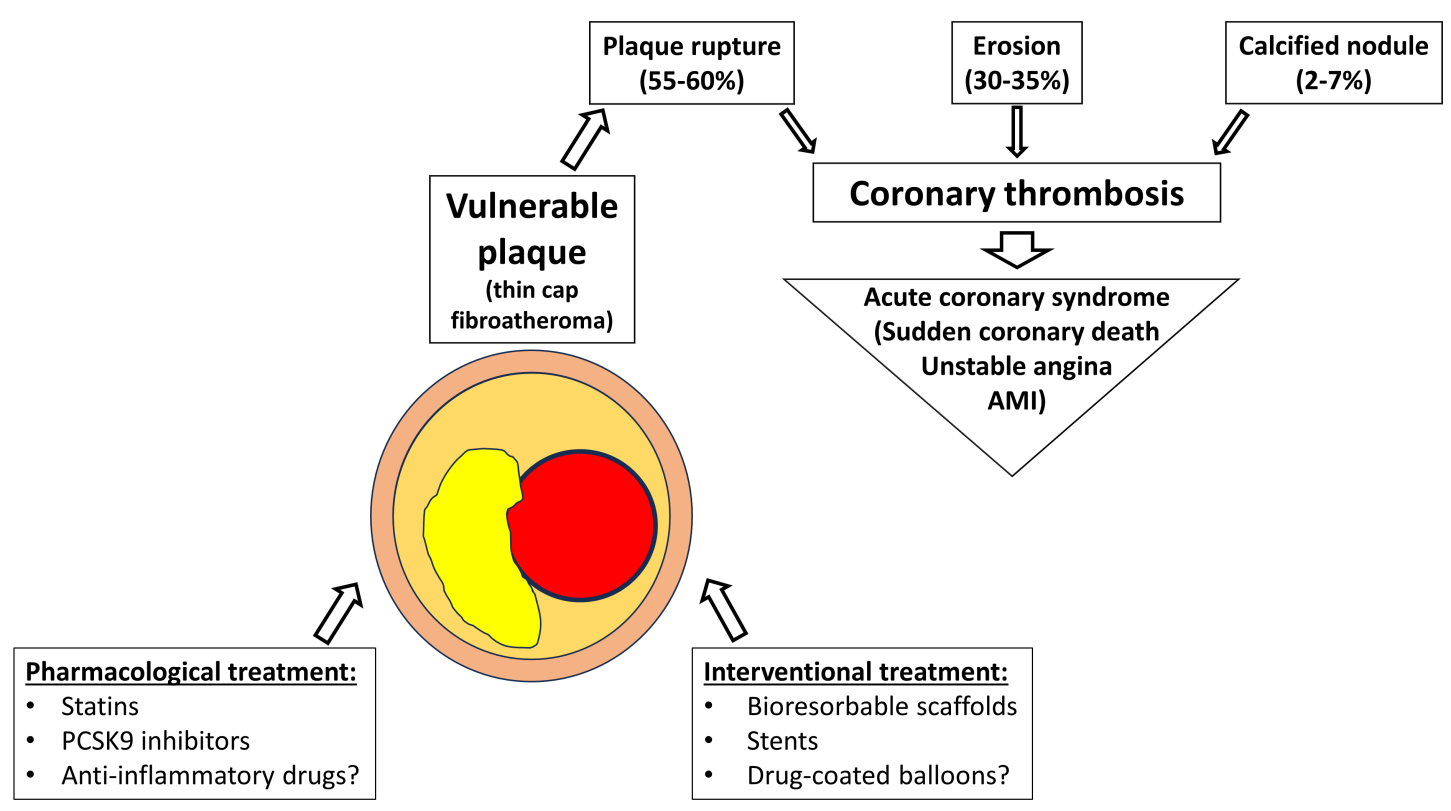
**Pathophysiology and therapeutic targets of a vulnerable plaque**. PCSK9, proprotein convertase subtilisin/kexin type 9; AMI, acute myocardial infarction.

Coronary atherosclerosis with the formation of lipidic plaques, whether flow-limiting or not, represents the most common underlying pathology of CVDs; atherosclerosis itself represents a multifactorial disease, especially coronary heart disease [2]. As consistently shown by many studies, the leading cause of acute coronary syndromes (ACSs) is intraluminal thrombosis, which, in turn, develops in a pathological background characterized by plaque rupture, plaque erosion, or eruptive calcific nodules [3, 4]. Atherosclerotic plaque rupture represents the main stimulus of thrombi formation [5, 6]; indeed, it is responsible for intraluminal thrombi formation in 55–60% of cases, whereas plaque erosion accounts for 30–35% and calcified nodules for 2–7% (Fig. 1) [7]. Plaque rupture predominantly occurs among lesions characterized by thin-cap fibroatheromas (TCFAs). Recently, it has been highlighted that features such as fibrous cap thickness, necrotic core size, and positive remodeling are critical features characterizing TCFAs and rupture-prone lesions [4, 8]. Vulnerable plaques (VPs) are most commonly TCFAs with a fibrous cap thickness of less than 65 µm, with loss of smooth muscle cells, extracellular matrix, and inflammatory infiltrate. VPs usually cover a large necrotic and lipid core, and intraplaque hemorrhage from vasa vasorum and calcifications are often present [3, 9]. Traditionally located in proximal coronary segments, VPs present positive vessel remodeling (Glagov effect), characterized by an elevated plaque burden with limited reduction in vessel lumen. For this reason, at coronary angiography, VPs usually appear with mild to moderate stenosis. However, not all thrombi developing on ruptured plaques lead to a clinical event because a non-occlusive thrombus can spontaneously heal, leading to the defined healed and layered plaques. Moreover, some studies showed that healed plaques, with the organization and stratification of residual thrombus, led to rapid plaque progression and further luminal narrowing [10, 11]. In a pooled analysis of two large databases, the prevalence of healed, layered plaques detected by optical coherence tomography (OCT) at the level of culprit lesions in patients with ACS was 29%; of these, about one-third presented a multilayered pattern, suggesting multiple, subclinical episodes of plaque rupture/thrombosis [12].

## 2. Invasive Assessment of Vulnerable Plaques

Several imaging modalities can be used to assess VPs in an invasive fashion. Each is particularly suited to evaluating one or more anatomical features.

### 2.1 Intravascular Ultrasound (IVUS)

Greyscale IVUS (GS-IVUS) is characterized by a high penetration power and, in the absence of thick arcs of calcium, can image all the coronary vessels from the lumen to the adventitia. Both minimal lumen area (MLA) and plaque burden can be easily calculated: Plaque burden (%) is defined as the external elastic membrane cross-sectional area minus the luminal cross-sectional area, then divided by the external elastic membrane cross-sectional area. Nowadays, high-resolution 60 MHz transducers are available, providing a spatial resolution of 22 microns and a penetration depth of 6 mm. However, despite these technical advancements, IVUS remains a suboptimal tool for evaluating plaque components, such as lipid core and macrophage infiltrates, and accurately identifying and measuring fibrous cap thickness [13, 14, 15].

### 2.2 Virtual Histology IVUS (VH-IVUS)

VH-IVUS represents an evolution of conventional GS-IVUS. VH-IVUS can identify four different components of coronary plaques through spectral analysis of the radiofrequency ultrasound signals: fibrous tissue, fibrofatty tissue, necrotic core, and calcium [16, 17]. Similarly to GS-IVUS, VH-IVUS is limited by low spatial resolution in the evaluation of fibrous cap; therefore, in the PROSPECT study, a thin-cap fibrous atheroma was defined according to the presence or absence of necrotic tissue encroaching the lumen [18]. However, while VH-TCFA was an independent predictor of recurrent events in the PROSPECT study, this was not confirmed in the ATHEROREMO-IVUS study. This may be due to the aforementioned limitations in spatial resolution, the acoustical shadowing of calcium, and the overlap between spectra identifying calcium and the necrotic core [19, 20].

### 2.3 Near-Infrared Spectroscopy IVUS (NIRS-IVUS)

Near-infrared spectroscopy, coupled with IVUS imaging, is a particularly suitable tool for quantitatively evaluating the lipid content of plaques because it yields a chemogram in which the presence of lipid core plaques is represented with high spatial resolution. Whereas the chemogram provides a qualitative representation of the vessel wall, with the lipid core depicted in yellow, the lipid-core burden index (LCBI) quantitatively evaluates the lipid content; thus, it has been extensively used in clinical studies [21]. The LCBI is the fraction of pixels with a probability of lipid greater than 0.6 divided by all analyzable pixels within the region of interest, multiplied by 1000. The LCBI is usually calculated on a chemogram derived from a region of interest, usually a 4 mm catheter pullback length; in this case, LCBI_4⁢m⁢m_ values range from 0 to 1000 [21, 22].

### 2.4 OCT

Presently, OCT is the best imaging technique for identifying TCFAs. OCT is a light-based imaging modality that generates a cross-sectional image of the vessel wall at a higher resolution (10 to 15 µm) than IVUS. However, a slight disadvantage of OCT is a minor tissue penetration depth of approximately 2 to 4 mm and the necessity of a bloodless field of view acquired with a continuous contrast liquid flush [23]. High-resolution OCT images can provide useful information about lipid-rich plaques, usually described as lipid arc circumferential extension, and deposits of calcium, which, differently from IVUS, can be penetrated by light, clearly representing their position and thickness. Moreover, OCT is the only technique able to evaluate the presence of activated macrophages, which reflect inflammation and appear as bright spots with a high signal attenuation behind [5, 24, 25].

### 2.5 Coronary Radial Wall Strain 

Coronary radial wall strain is a novel diagnostic modality assessing vessel wall deformation during the cardiac cycle. This is an angiography-derived parameter acquired through the analysis of two different angiographic views of the same vessel without overlapping or through a single angiographic projection with the help of artificial intelligence calculations. Coronary radial wall strain appears to be a promising new technique because different wall/plaque features have different stiffnesses, thus affecting strain values [26, 27].

## 3. Noninvasive Assessment of Vulnerable Plaques

Cardiac computed tomography angiography (CCTA) can identify coronary stenosis with a high sensibility and sensitivity, and it is being widely used as a diagnostic tool for coronary artery disease in high-risk patients [28, 29]. CCTA can also detect several plaque characteristics typical of a VP phenotype, such as positive remodeling, spotty calcification, napkin-ring images, and low attenuation plaque value [30]. Several studies investigated the association of such high-risk plaque (HRP) characteristics with coronary events. For the first time, Otsuka *et al*. [31] reported that the napkin-ring sign, defined as a plaque core of low attenuation surrounded by a ring of higher attenuation, was a predictor of ACS events independent of other HRP characteristics. Motoyama *et al*. [30] reported that HRP characteristics detected by CCTA were independent predictors of ACS development over a mean follow-up of 3.9 years. Motoyama and co-authors also observed that the plaque progression in patients with serial CCTA scans strongly predicted major adverse events [32]. A secondary analysis of the PROMISE trial data showed that patients who had HRP features on CCTA scans had a much higher major adverse cardiovascular event (MACE) rate, with a hazard ratio of 2.73 (95% CI 1.89–3.93), independent of the presence of severe artery stenosis and atherosclerotic cardiovascular disease risk score [33]. However, Ferencik *et al*. [34] underlined that a low absolute rate of MACEs determined a low positive predictive value, therefore questioning if detecting high-risk plaques using CCTA could have a significant role in clinical practice [32]. A post-hoc analysis of the SCOT-HEART study showed that low-attenuation plaque burden was the strongest predictor of myocardial infarction in patients with stable chest pain at a median follow-up of 4.7 years [35]. In 2024, an observational, prospective cohort study enrolled patients with chronic coronary syndromes and non-ST-elevation ACS and investigated the concordance between HRP features detected by CCTA and TCFAs detected by OCT. This study showed that all HRP characteristics, with positive remodeling being the most prevalent, were associated with TCFAs (the more HRP features were present, the higher the association with TCFA lipid-rich plaques (LRPs) and macrophage presence) and that untreated HRPs in culprit vessels were at higher risk of developing cardiovascular adverse events [36]. Artificial intelligence could represent a useful tool in the multiparametric evaluation of coronary plaques to establish features predictive of adverse events. This approach has been tested in the EMERALD-II study, in which artificial intelligence—enabled quantitative plaque and hemodynamic analysis—was conducted on previously performed computed tomography (CT) scans in patients undergoing coronary angiography for ACS [37]. Compared to a reference model based on conventional HRP characteristics, such as plaque burden, low attenuation plaques, and napkin ring sign, the model based on artificial intelligence analysis incorporated flow dynamics and showed a higher predictive power for adverse events.

Cardiac magnetic resonance imaging is another noninvasive imaging modality that can detect VPs, which appear as high-intensity plaques with a plaque-to-myocardial signal intensity ratio >1.4. This plaque phenotype has been associated with future coronary events [38] and OCT-derived characteristics of HRPs, such as LRPs, macrophage accumulation, and healed plaque ruptures [39]. However, prognostic data from magnetic resonance imaging studies are still limited compared to data derived from CCTA studies.

A new multimodality imaging database (NCT04523194) was recently established that incorporates CCTA scans, OCT images, coronary angiogram acquisitions, and cardiac magnetic resonance images. This multimodality approach has the potential to help better define the prognostic and pathophysiological role of VPs.

## 4. Association of VPs with Adverse Events: Observational Studies

The PROSPECT trial was the first study to investigate the natural history of non-culprit coronary plaques characterized by the greyscale- and VH-IVUS during percutaneous coronary intervention (PCI) of culprit lesions in 697 patients with ACS [18]. Non-culprit lesions leading to future adverse events were angiographically mild (32.3 ± 20.6% mean diameter stenosis) at baseline; high-risk plaque characteristics (plaque burden ≥70%, MLA ≤4.0 mm^2^, and the presence of TCFAs) were associated with higher MACE rates at the 3-year follow-up. The LRP study used NIRS-IVUS to detect lipid content in non-culprit coronary plaques in 1271 patients undergoing coronary angiography. The study aimed to investigate the correlation between lipid-rich plaques and the risk of MACEs at the 24-month follow-up [40]. At the 2-year follow-up, 9% of the study population suffered from MACEs related to the non-culprit lesions. Lesions with a maxLCBI_4⁢m⁢m_ ≥400 were independently associated with MACEs, irrespective of plaque burden and MLA, with an adjusted hazard ratio of 3.39 (95% CI 1.85–6.20; *p* < 0.0001). CLIMA was the first large prospective observational study to investigate the association of HRP characteristics detected by OCT with MACEs in a cohort of 1003 patients with coronary plaques involving the proximal segment of the left anterior descending coronary artery [41]. In this study, four main plaque characteristics (MLA <3.5 mmq, fibrous cap thickness <75 µm, lipid arc circumference >180° and presence of macrophage infiltration clusters) were found to be predictive of events at 1-year follow-up; the simultaneous presence of all these characteristics, although present in only 3.6% of patients, had the strongest predictive value for MACEs (HR 7.54, 95% CI 3.1–18.6; *p* < 0.001). The design of the PROSPECT II study was similar to the PROSPECT; however, NIRS-IVUS was used instead of VH-IVUS to investigate the association of plaque burden and lipid core index of non-culprit lesions with MACEs at follow-up [42]. Similarly to the PROSPECT study, the mean baseline angiographic diameter stenosis of untreated non-culprit lesions that were subsequently associated with MACEs was not significant (46.9%); the presence of both large plaque burden and high lipid content was the stronger predictor of events with an odds ratio (OR) of 11.33 (95% CI 6.10–21.03). The COMBINE OCT-fractional flow reserve (FFR) study focused on patients with diabetes mellitus; in this study, the presence of TCFAs detected by OCT in FFR-negative coronary lesions was associated with a five-fold higher rate of MACEs at 18 months follow-up as compared to patients without such plaque feature [43]. Subsequently, a further study from the same group showed that the simultaneous presence of TCFAs and LRPs, assessed by extension of lipid arc at OCT, was associated with a higher rate of events as compared to LRPs with TCFAs or non-LRPs [43]. Recently, 5-year follow-up data were published, confirming the predictive value of OCT-detected TCFAs in this subset of patients [44]. The predictive role of TCFA and MLA <3.5 mm^2^ was confirmed by Jiang *et al*. [45], who performed OCT evaluation of all three main epicardial arteries in 883 patients with acute MI during primary PCI [46]. In the multivariable analysis in this study, the hazard ratio for MACEs at the lesion level at 3.3 years follow-up was 15.50 (95% CI 6.89–34.89; *p* < 0.001) for plaques with both high-risk characteristics. Intriguing findings on the interplay between coronary physiology and the presence of VP detected by OCT or CT were recently reported in a subanalysis of the P3 (Precise Percutaneous Coronary Intervention Plan) study [47]. In patients with chronic coronary syndromes and coronary lesions with FFR values <0.80 [48], the authors observed that lower FFR and higher pullback pressure gradient values (the latter identifying focal pattern of coronary atherosclerosis) were the strongest predictors of the presence of VPs, possibly as a consequence of increased shear stress at the plaque level.

## 5. Pharmacological Treatment of VPs

Increasing evidence shows that lipid-lowering therapy can significantly reduce the volume of coronary plaques and induce changes in their composition, with an increase in the fibrous cap and reduction in the lipid pool; this was observed in several randomized trials conducted with statins [49, 50]. The introduction of proprotein convertase subtilisin/kexin type 9 (PCSK9) inhibitors and, more recently, short interfering RNA (siRNA) has further increased the possibility of reaching target low-density lipoprotein (LDL) blood levels (<55 mg/dL according to international guidelines) even in patients with statin intolerance. Similar to what was observed with statins, several randomized trials also showed a significant impact of PCSK9 inhibitors on plaque volume and composition. In the PACMAN-AMI trial, the addition of alirocumab on top of high-intensity statin therapy (rosuvastatin 20 mg) led to a reduction in atheroma volume and lipid content, assessed by NIRS-IVUS and an increase in fibrous cap thickness, assessed by OCT at the level of non-culprit, non-obstructive coronary lesions in patients with acute MI as compared to placebo [51]. Consistent results were observed in the HUYGENS trial, in which evolocumab, on top of high-intensity statin therapy, led to similar results in the primary endpoint (increase in fibrous cap thickness) and secondary endpoint (reduction in maximum lipid arc assessed by OCT) [52]. Interestingly, the changes in plaque composition in both studies were mirrored by the changes in LDL blood levels, which were, as expected, lower in the PCSK9 arm, suggesting a “lower is better” effect regarding plaque regression and stabilization.

The beneficial effect of PCSK9 inhibitors could also be mediated by reducing blood levels of lipoprotein(a) [53], which is currently being investigated as a potential therapeutic target for cardiovascular prevention. High levels of lipoprotein(a) were indeed reported to be associated with the progression of low attenuation (necrotic core) plaques assessed by serial CT examinations [54].

Another intriguing secondary prevention strategy is represented by anti-inflammatory therapy. Indeed, inflammation is a well-known key component in both coronary atherosclerosis and coronary plaque destabilization [55]; several biomarkers detected in peripheral blood [56], such as high sensitivity C-reactive protein (hs-CRP), plasminogen activator inhibitor-1, fibrinogen, interleukin-6, homocysteine and amyloid A were found to accurately predict the presence of vulnerable, high-risk plaques defined according to IVUS and OCT criteria [57]. Based on this background, the CANTOS trial finally demonstrated the inflammatory hypothesis, in which patients with a history of myocardial infarction and elevated levels of hs-CRP were randomized to different doses of kanakinumab, an interleukin-6 inhibitor or placebo. The trial showed that the hs-CRP and interleukin-6 levels were significantly reduced in the intervention group compared to placebo; this was also associated with a reduction in the incidence of MACEs at follow-up in the 150 mg canakinumab group [58]. Following the results of the CANTOS trial, two other randomized trials yielded similar results with the administration of colchicine. However, whether anti-inflammatory therapy may stabilize high-risk coronary plaques is not known at present; an ongoing randomized trial, the COCOMO-ACS, is investigating whether colchicine at 0.5 mg/day in patients with a recent myocardial infarction can increase the fibrous cap thickness at an 18-month follow-up, compared to a placebo [59]. In conclusion, several drugs, through their hypolipemic and anti-inflammatory properties, have the potential to stabilize VPs, thus reducing future coronary events; given the systemic nature of atherosclerotic disease, such a strategy could have a greater impact and be more cost-effective as compared to a strategy of preventive PCI, which is characterized by a focal rather than systemic treatment.

## 6. Preventive PCI of VPs: Randomized Trials

**Fig. 2.**
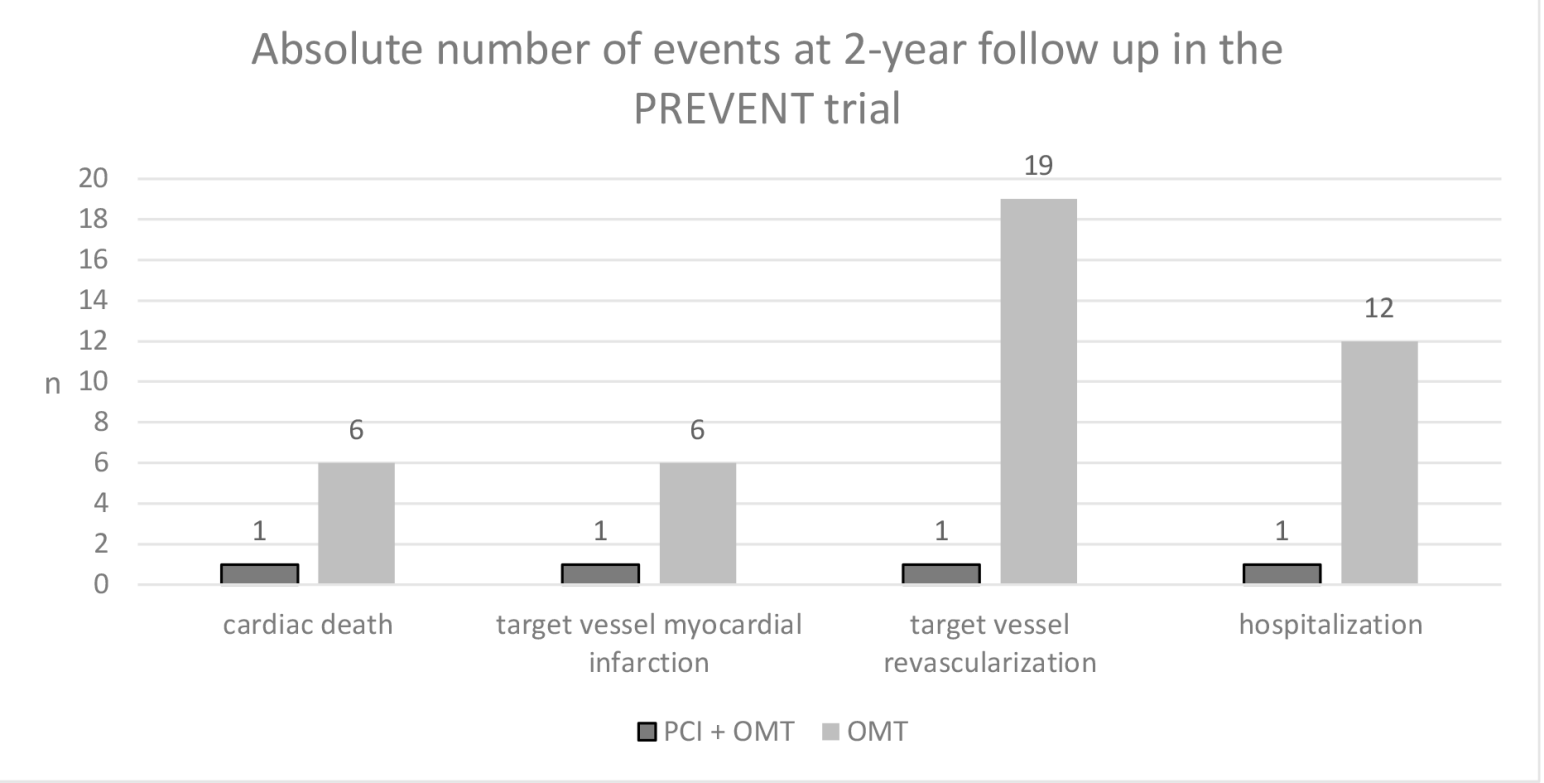
**Components of the primary endpoint in the Preventive Coronary Intervention on Stenosis with Functionally Insignificant Vulnerable Plaque (PREVENT) trial**. PCI, percutaneous coronary intervention; OMT, optimal medical therapy.

In recent decades, interest in developing interventional strategies for passivating VPs to prevent coronary events has increased. The first of these studies, the SECRITT, was published in 2012 [60]. In this pilot, proof-of-concept trial, 23 patients with non-obstructive, vulnerable lesions, defined as TCFAs according to VH-IVUS and OCT criteria, were randomized to PCI following the implantation of a self-expanding, nitinol stent (n = 13) or conservative treatment. The primary endpoint of the study was the change in vessel wall strain as assessed by IVUS palpography; the strain is higher in LRPs than in fibrous or calcified plaques. A significant decrease in vessel wall strain was detected in the stenting group. Similarly, a significant increase in cap thickness at 6-month follow-up was also observed. Although not powered for clinical events, the SECRITT trial suggested that plaque sealing with a stent could be an effective strategy to reduce the lipid content of the plaque and increase the cap thickness. The PROSPECT-ABSORB was the first large trial comparing an interventional and a conservative approach to managing high-risk, non-obstructive plaques [61]. This study enrolled patients with acute myocardial infarction (MI) after successful PCI of the culprit and other significant lesions based on the intracoronary pressure wire study result. Non-obstructive lesions with plaque burdens ≥65% at IVUS could be randomized to implantation of a coronary bioresorbable vascular scaffold (BVS) (Absorb, Abbott Vascular, Santa Clara, CA, USA) or conservative treatment. IVUS imaging was coupled with NIRS to assess the LCBI, but the operators were blinded to NIRS data. In total, 182 patients were randomized to scaffold implantation (n = 93) or conservative treatment (n = 89); the primary endpoint, the IVUS-derived MLA, was significantly higher in lesions undergoing interventional therapy as compared to controls (6.9 ± 2.6 mm^2^ vs. 3.0 ± 1.0 mm^2^; *p* < 0.0001). No difference was observed in the rate of target lesion failure and MACEs at follow-up, but the study was not powered for clinical events. Notably, only about 40% of randomized lesions presented all NIRS-IVUS characteristics that were used to define high-risk plaque morphology: (1) plaque burden ≥70% (the only NIRS-IVUS criterium for eligibility), (2) maxLCBI >324.7, and (3) MLA ≤4.0 mm^2^. Interestingly, the maxLCBI was significantly reduced at follow-up in the interventional group as compared to the control group (62 [0.0, 213.8] vs. 268.8 [157.2, 396.7]; *p* < 0.0001). Although powered for a surrogate endpoint and not for clinical endpoints, the PROSPECT-ABSORB trial showed that PCI of angiographically intermediate, functionally not significant lesions is safe and is associated with increasing MLA at follow-up. Indeed, this finding was despite using a device later associated with a higher rate of target vessel failure (TVF) than metallic drug-eluting stent (DES). The first large trial of preventive PCI of VPs powered for clinical events was recently published [62]. In the PREVENT trial, 1800 patients, mostly presenting with chronic coronary syndromes, after successful PCI of functionally significant coronary lesions, were randomized 1:1 to conservative treatment or PCI with BVS or DES (following the withdrawal from the market of the Absorb device) of vulnerable coronary plaques defined as follows: (1) diameter stenosis >50% at angiography; (2) FFR >0.80; MLA-IVUS <4.0 mm^2^; (3) IVUS plaque burden >70%; (4) LRP on NIRS; (5) TCFA by OCT or VH-IVUS. The incidence of the primary endpoint of the study, TVF at 2 years, was 3.4% in the conservative and 0.4% in the interventional group (HR 0.54, 95% CI 0.33–0.87). Although the results of the PREVENT trial favored an interventional approach to VPs, several issues must be discussed. First, the rate of events in both groups was lower than expected; indeed, in the control group, the rate of TVF was 3.4% at 2 years, similar to that observed in the FAME 2 registry, which enrolled patients with functionally non-significant (FFR >0.80) coronary lesions treated conservatively [63]. This suggests that leaving non-significant coronary lesions, especially in patients with predominantly stable coronary artery disease, represents a reasonable strategy. Indeed, if we look at the single components of the combined primary endpoints in the PREVENT study, we can appreciate that the benefit of PCI is mostly driven by the reduction in target vessel revascularization (TVR) and rehospitalizations, whereas the number needed to treat and prevent hard endpoints (the composite of cardiac death and target vessel myocardial infarction) is 87.7 (Fig. 2).

Furthermore, TVR and rehospitalization could have been inflated in the control group because of the open-label design of the study, in which both the patient and the doctor were aware of the type of treatment performed or, better yet, of the fact that a potentially risky plaque had not been treated. Other relevant issues in the study were related to pharmacological treatment. Double antiplatelet therapy (DAPT) was obviously administered longer in the PCI groups compared to the control group; at one year, 48% of PCI patients were still on DAPT compared to 23% of controls. Given the well-known role of DAPT in preventing MACEs, a bias favoring the invasive strategy cannot be ruled out. Regarding cholesterol-lowering therapy, it should be noted that mean LDL cholesterol levels in both groups were around 64 mg/dL, which suggests a suboptimal LDL control according to the recommendations of international guidelines in patients with coronary artery disease; therefore, the trial is not informative regarding the additional benefit of preventive PCI (eventually on top of optimal lipid-lowering therapy) as compared to lipid-lowering therapy alone. In conclusion, although the authors of the PREVENT study should be congratulated for providing the first large trial powered for clinical endpoints that provide evidence in favor of the interventional treatment of VPs, further data are eventually needed to recommend this strategy.

### Ongoing Clinical Trials

A synopsis of the ongoing clinical trials is reported in Table 1. The COMBINE-INTERVENE study (NCT05333068) compares a revascularization strategy based on the combined use of OCT and FFR with a strategy based solely on FFR in a population of patients with multivessel coronary artery disease (luminal stenosis of at least 50%) who are candidates for PCI, regardless of clinical presentation. In the OCT–FFR group, lesions were treated if they had an FFR ≤0.75 or if OCT revealed the presence of a TCFA (cap thickness ≤0.75 microns), plaque rupture, or plaque erosion; in the FFR group, lesions were treated if they had an FFR ≤0.80. The study was single-blind, as patients randomized to the FFR arm underwent a sham OCT procedure. The primary endpoint of the study was a composite of cardiac death, myocardial infarction, and revascularization at 2 years. The EPIC28-VULNERABLE study (NCT05599061) also considered ST-elevation myocardial infarction (STEMI) patients with intermediate-grade multivessel disease (40–69%), negative FFR, and VP characteristics on OCT evaluation; patients were randomized to PCI with everolimus-eluting stents and optimal medical therapy or to optimal medical therapy alone. The primary endpoint was TVF (a composite of cardiovascular death, myocardial infarction, and revascularization) at 4 years. In our view, this study is the best designed, as it requires FFR assessment in all patients before randomization, directly comparing interventional treatment with conservative management of VP. The FAVOR V study (NCT05669222), following the line of previous FAVOR studies, is characterized by the use of noninvasive methods based on advanced computerized angiography analysis (FAST: functional and angiography-derived strain integration) for both functional assessment (next-generation quantitative flow ratio (µQFR)) and plaque vulnerability evaluation (radial wall strain). The study targets patients with STEMI undergoing primary PCI and multivessel disease (non-culprit stenosis: 50–90%), comparing a revascularization strategy guided by FAST criteria (µQFR ≤0.80 or RWS ≥13%) with a PCI strategy for angiographic stenosis ≥70% or for intermediate angiographic stenosis (50–70%) with significant fractional flow reserve /instantaneous wave-free ratio (FFR/iFR) values. The primary endpoint of the study was a composite of death, myocardial infarction, and ischemia-driven revascularization. The INTERCLIMA study (NCT05027984) targets patients with ACS and multivessel coronary artery disease with non-culprit lesions of intermediate severity (40–70% on quantitative coronary angiography (QCA)). Moreover, the study compares an OCT-guided revascularization strategy (indication for PCI if a fibrous cap thickness <75 microns is present along with at least two additional criteria among MLA <3.5 mm^2^, lipid arc with circumferential extension >180°, and presence of macrophages) with a physiology-guided strategy (resting index ≤0.89 or FFR ≤0.80). The primary endpoint of the study was ambitious, comprising a composite of cardiac death and myocardial infarction at 2 years of follow-up.

**Table 1.** **Ongoing randomized clinical trials on preventive PCI of vulnerable plaques**.

Clinicaltrials.gov ID	Acronym	Clinical setting	Experimental arm	Comparator arm	Primary outcome
NCT05333068	COMBINE INTERVENE	Both acute and chronic coronary syndromes; multivessel CAD undergoing PCI	FFR (≤0.75) and OCT (TCFA, ruptured plaque, plaque erosion) guided PCI	FFR (≤0.80)-guided PCI	Cardiac death, any MI, or any clinically-driven revascularization at 24 months
NCT05027984	INTERCLIMA	Intermediate (40–70% DS), non-culprit coronary lesions in ACS patients undergoing coronary angiography	OCT guided PCI (FCT <75 µm, MLA <3.5 mm^2^, lipid arc >180°, macrophages)	FFR (iFR, RFR)-guided PCI	Cardiac death or non-fatal spontaneous target-vessel MI at 24 months
NCT05599061	VULNERABLE	Intermediate (40–69% DS) FFR- non-culprit lesions with features of vulnerable plaques in OCT in MV STEMI patients	PCI with EES + OMT	OMT	TVF at 4 years (cardiovascular death, target-vessel related MI, clinically, and physiologically oriented TVR)
NCT05669222	FAVOR V AMI	Intermediate (50–90% DS) non-culprit lesions in MV STEMI patients	µQFR or RWS- guided PCI	Angio (DS ≥70%) or FFR/iFR-guided PCI (DS 50–70%)	All-cause death, MI, or ischemia-driven revascularization at 1.5 years

CAD, coronary artery disease; PCI, percutaneous coronary intervention; OCT, optical coherence tomography; FFR, fractional flow reserve; iFR, instantaneous wave-free ratio; RFR, resting full-cycle ratio; TCFA, thin cap fibroatheroma; MI, myocardial infarction; DS, diameter stenosis; ACS, acute coronary syndrome; FCT, fibrous cap thickness; MLA, minimal lumen area; MV, multivessel; STEMI, ST-elevation myocardial infarction; TVF, target vessel failure; TVR, target vessel revascularization; µQFR, next-generation quantitative flow ratio; RWS, radial wall strain; EES, everolimus-eluting stent; OMT, optimal medical therapy.

## 7. Challenges and Future Perspective of Preventive PCI

Despite its limitations, the results of the PREVENT study showed for the first time that preventive PCI can reduce cardiac events compared to placebo. However, several issues must be considered before recommending the widespread adoption of such a strategy.

First, although several observational studies with different imaging techniques consistently showed that VPs are associated with an increased risk of adverse events at follow-up, the overall predictive value is low; for example, the incidence of plaque-specific adverse coronary events at follow-up was 4.3% in PROSPECT (26 events in 596 TCFA) and 4.1% in SCOT-HEART (25 events in 608 coronary plaques with positive remodeling or low attenuation). Accordingly, in a large meta-analysis evaluating the association of several coronary plaque characteristics detected with intracoronary imaging (high plaque burden, low MLA, TCFA, large lipid core burden index) or with CT scan (low attenuation plaque, positive remodeling, napkin-ring sign, spotty calcification) the positive predictive value ranged from 8 to 24; even when considering the presence of at least two plaque characteristics, the PPV was 22 (95% CI 15–27) [64]. The absolute low rate of the event despite high-risk plaque characteristics can be explained by the dynamic nature of VPs, which may either undergo subclinical episodes of rupture and thrombosis, eventually leading to the layered phenotype [12], or simply heal, as shown in a study by Kubo *et al*. [65]. Here, Kubo *et al*. [65] performed serial VH-IVUS of TCFAs and observed that 75% of lesions changed their phenotype (mostly becoming thick cap fibroatheroma), whereas the others remained unchanged. Interestingly, healing was not related to lipid-lowering therapy or changes in LDL blood levels, suggesting that most VPs can heal irrespective of active therapeutic strategies. Second, MLA appeared to be one of the high-risk plaque characteristics in several studies, such as PROSPECT and CLIMA. Differently from the presence of a thin cap underlying a large amount of necrotic tissue or lipid pool, from a pathophysiological point of view, it is unclear why a reduced MLA should be predictive of adverse coronary events, especially of coronary rupture and thrombosis unless a reduction in coronary reserve is hypothesized. Indeed, the FAME 2 trial showed that not performing PCI of FFR-positive lesions leads to an increased rate of adverse coronary events (mostly TVR) at follow-up. Indeed, FFR evaluation was not performed in both the PROSPECT and CLIMA studies; moreover, MLA cutoffs in these studies (≤4.0 mm^2^ and ≤3.5 mm^2^, respectively) are close to the value of 3.1 mm^2^, which was recently shown to be predictive of an FFR <0.80 in proximal coronary segments, as were those included in the CLIMA study [66]. Therefore, the possibility that a proportion of coronary events (especially TVRs) in observational studies on VPs might be secondary to functionally significant lesions left untreated cannot be ruled out. Nevertheless, we must acknowledge that, in the PREVENT study, although an MLA <4 mm^2^ was one of the qualifying criteria, only non-flow limiting (FFR >0.80) coronary VPs were considered; however, this could partially explain the lower-than-expected rate of events in this trial.

Third, the economic costs, procedural length, and risk of complications of a preventive PCI strategy, including multivessel (and multimodality) coronary imaging, should be weighed against clinical benefit. Indeed, despite tremendous improvement in devices and drug therapy, stenting can still be associated with “an iatrogenic disease”, considering the 2.2% rate of target vessel myocardial infarction at 1 year [67]. Notably, as highlighted in an editorial by Johnson *et al*. [68], any strategy of preventive PCI is by default associated with an immediate 100% TVF rate because every patient undergoes PCI of a non-culprit, functionally non-significant lesion because of its high-risk characteristics; in a hypothetical control arm, in which PCI does not treat such lesions, the risk of TVF, albeit high, would hardly approach this figure. Moreover, even if we approximate the risk of in-stent restenosis or stent thrombosis to zero, PCI of VPs may be associated with an increased risk of peri-procedural MI and no-reflow. Indeed, it has been shown that both events, possibly due to distal embolization of plaque material, were significantly more frequent after stenting of lipid-rich plaques detected using NIRS-IVUS compared to plaques with lower lipid content [69, 70]. This issue must be acknowledged in the analysis of the risk–benefit ratio of preventive PCI because large periprocedural MIs (with a rise in cardiac biomarkers >5 times the upper reference limit) are associated with increased risk of all-cause and cardiac mortality at follow-up [71].

Finally, the potential benefit of preventive PCI is also dependent on the absolute risk of the population; in patients at high risk, the benefit of preventive PCI likely outweighs both the peri-procedural risk and the risk of stent-related adverse events (thrombosis and restenosis) in the medium to long term, whereas this might not apply to patients at low to intermediate risk.

The role of preventive PCI in particular subsets of patients, such as older adults, is even more elusive. Recently, the FIRE trial showed that a strategy of complete revascularization guided by fractional flow reserve was superior to a conservative approach in which only the culprit lesion was treated in patients aged ≥75 years presenting with an ACS [72]. Differently, evidence on treating VPs in these patients is scarce; indeed, the average age of enrolled patients was around 65 years in PROSPECT-ABSORB and PREVENT studies. Theoretically, the risk/benefit ratio of preventive PCI in older adults should be less favorable compared to younger patients because older patients suffer from a higher rate of peri-procedural complications [73] and may benefit less from the prevention of future coronary events due to their reduced life expectancy.

Another issue is the long-term benefit of preventive PCI. The DEFER study showed that the prognosis of medically treated, FFR-negative coronary stenosis remains good for up to 15 years [74]. Conversely, stented lesions show an ongoing, albeit low, rate of events, especially type 1 myocardial infarction, without evidence of a plateau [67].

Accordingly, the drawbacks of implanting a metallic stent to seal and passivate VPs have been clear to the investigator since the initial studies. Indeed, a bioresorbable vascular scaffold (Absorb, Abbott Vascular, Santa Clara, CA, USA) was selected in both the PROSPECT-ABSORB and in the initial phase of the PREVENT; however, this is no longer available due to suboptimal performance as compared to a metallic DES [75]. Meanwhile, drug-coated balloons represent another possible strategy to avoid a permanent implant. A recent pilot study showed that PCI of lipid-rich plaques, identified by IVUS-NIRS, with a paclitaxel-coated balloon was associated with a significant reduction in maxLCBI_4⁢m⁢m_ at a 9-month follow-up [76]. However, whether this strategy could have a role in managing VPs needs to be evaluated in larger clinical trials.

## 8. Conclusions

Preventive PCI of vulnerable coronary plaques may represent a useful strategy to reduce first or recurrent cardiovascular events in addition to optimal medical therapy. However, given the low predictive value of high-risk plaque characteristics and the dynamic nature of VPs, which often heal and stabilize independent of active treatments, this strategy should be directed at patients at very high risk, such as patients with ACS, who present an event rate at follow up significantly higher than patients with chronic coronary syndromes. Accordingly, most ongoing randomized trials (INTERCLIMA, VULNERABLE, FAVOR V) are focused on patients with STEMI and multivessel coronary artery disease, a high-risk clinical setting in which a strategy of preventive PCI was initially evaluated in the PRAMI trial [77]. In addition to clinical and coronary plaque characteristics, lipoprotein(a) blood level measurement could be useful in selecting a high-risk population. Elevated lipoprotein(a) levels were found to be associated with a higher prevalence of lipidic plaques and TCFA in coronary lesions [78] and a high rate of progression and vulnerability in carotid lesions [79]. In a selected population of patients with acute myocardial infarction treated by PCI, we observed that elevated lipoprotein(a) levels at admission were associated with an increased rate of MACEs at follow-up (HR 1.82 [95% CI 1.04–3.19]) [80]. Whether preventive PCI of VPs may be a reasonable strategy to reduce recurrent events will be assessed by ongoing randomized trials; in our opinion, the VULNERABLE trial (NCT05599061) could be particularly informative to appraise the efficacy of preventive PCI because this study compares preventive PCI alongside optimal medical therapy with optimal medical therapy alone.

Even in the best scenario, we should remember that preventive PCI would be ineffective against the other pathological substrates of coronary thrombosis, namely plaque erosion and calcific nodules, which account for about 40% of cases. We believe no clinical indication exists to identify and treat such lesions outside of a randomized study. Clinicians should instead pay greater attention to lipid-lowering therapies to achieve LDL cholesterol targets.

## Abbreviations

CVD, cardiovascular disease; ACS, acute coronary syndrome; TCFA, thin cap fibroatheroma; VP, vulnerable plaque; OCT, optical coherence tomography; GS-IVUS, greyscale intravascular ultrasound; MLA, minimal lumen area; VH, virtual histology; NIRS, near infrared spectroscopy; LCBI, lipid core burden index; CCTA, cardiac computed tomography angiography; HRP, high risk plaque; MACE, major adverse cardiovascular events; PCI, percutaneous coronary intervention; PCSK9, proprotein convertase subtilisin/kexin type 9; siRNA, short interfering RNA; TVF, target vessel failure; DES, drug eluting stent; TVR, target vessel revascularization; DAPT, double antiplatelet therapy.
